# Poly[[penta­aqua­(μ_4_-pyridine-2,4,6-tri­carboxyl­ato)(μ_3_-pyridine-2,4,6-tri­carboxyl­ato)disamarium(III)] mono­hydrate]

**DOI:** 10.1107/S1600536812002462

**Published:** 2012-02-10

**Authors:** Yong-Wei Jin, Hong-lin Zhu

**Affiliations:** a Crystal Engineering Division, Center of Applied Solid State Chemistry Research, Ningbo University, Ningbo, Zhejiang 315211, People’s Republic of China

## Abstract

The asymmetric unit of the title compound, {[Sm_2_(C_8_H_2_NO_6_)_2_(H_2_O)_5_]·H_2_O}_*n*_, contains two independent Sm^III^ ions, two pyridine-2,4,6-tricarboxyl­ate (ptc) ligands, five aqua ligands and one lattice water mol­ecule. One Sm^III^ ion is nine-coordinated by one N and five O atoms from the three ptc ligands and three aqua ligands in a distorted monocapped square antiprismatic geometry, and the other is eight-coordinated by one N and five O atoms from three ptc ligands and two aqua ligands in a 4,4′-bicapped trigonal anti­prismatic geometry. The ptc ligands brigde the Sm^III^ ions into a three-dimensional polymeric framework. Extensive O—H⋯O hydrogen bonding is observed in the crystal structure.

## Related literature

For related compounds, see: Gao *et al.* (2006 ▶); Ghosh & Bharadwaj (2005 ▶); Li *et al.* (2008 ▶); Wang *et al.* (2007 ▶).
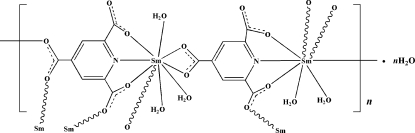


## Experimental

### 

#### Crystal data

[Sm_2_(C_8_H_2_NO_6_)_2_(H_2_O)_5_]·H_2_O
*M* *_r_* = 825.01
Monoclinic, 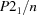

*a* = 18.426 (4) Å
*b* = 6.9082 (14) Å
*c* = 18.583 (4) Å
β = 111.98 (3)°
*V* = 2193.6 (8) Å^3^
*Z* = 4
Mo *K*α radiation
μ = 5.40 mm^−1^
*T* = 293 K
0.43 × 0.28 × 0.21 mm

#### Data collection

Rigaku R-AXIS RAPID diffractometer
Absorption correction: multi-scan (*ABSCOR*; Higashi, 1995 ▶) *T*_min_ = 0.177, *T*_max_ = 0.321
20300 measured reflections
4963 independent reflections
4776 reflections with *I* > 2σ(*I*)
*R*_int_ = 0.035

#### Refinement

*R*[*F* ^2^ > 2σ(*F* ^2^)] = 0.018
*wR*(*F* ^2^) = 0.041
*S* = 1.19
4963 reflections
344 parameters
H-atom parameters constrained
Δρ_max_ = 0.69 e Å^−3^
Δρ_min_ = −0.84 e Å^−3^

### 

Data collection: *RAPID-AUTO* (Rigaku, 1998 ▶); cell refinement: *RAPID-AUTO*; data reduction: *CrystalStructure* (Rigaku/MSC, 2004 ▶); program(s) used to solve structure: *SHELXS97* (Sheldrick, 2008 ▶); program(s) used to refine structure: *SHELXL97* (Sheldrick, 2008 ▶); molecular graphics: *SHELXTL* (Sheldrick, 2008 ▶); software used to prepare material for publication: *SHELXL97*.

## Supplementary Material

Crystal structure: contains datablock(s) global, I. DOI: 10.1107/S1600536812002462/cv5233sup1.cif

Structure factors: contains datablock(s) I. DOI: 10.1107/S1600536812002462/cv5233Isup2.hkl

Additional supplementary materials:  crystallographic information; 3D view; checkCIF report

## Figures and Tables

**Table 1:** Hydrogen-bond geometry (Å, °)

*D*—H⋯*A*	*D*—H	H⋯*A*	*D*⋯*A*	*D*—H⋯*A*
O7—H7*A*⋯O14^i^	0.82	1.87	2.686 (3)	172
O7—H7*B*⋯O2^ii^	0.86	1.80	2.645 (3)	165
O8—H8*A*⋯O12^iii^	0.89	1.83	2.727 (3)	177
O8—H8*B*⋯O9^iv^	0.87	2.38	2.831 (3)	113
O9—H9*A*⋯O6^i^	0.87	1.84	2.698 (3)	171
O9—H9*B*⋯O5^i^	0.93	2.55	3.052 (3)	114
O16—H16*A*⋯O4^i^	0.82	2.31	3.075 (3)	157
O16—H16*B*⋯O15^v^	0.79	1.97	2.747 (3)	171
O17—H17*A*⋯O18^vi^	0.84	1.86	2.697 (3)	176
O17—H17*B*⋯O5^vii^	0.80	1.96	2.731 (3)	162
O18—H18*A*⋯O14^viii^	0.85	2.06	2.905 (3)	174
O18—H18*B*⋯O13	0.82	1.93	2.736 (3)	168

Symmetry codes: (i) 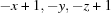; (ii) 
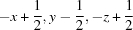
; (iii) 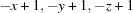; (iv) 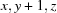; (v) 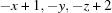; (vi) 
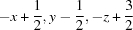
; (vii) 
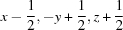
; (viii) 
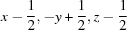
.

**Fig. 1.**
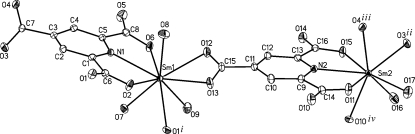
A portion of the crystal structure showing the atomic numbering and 45% probability dispalcement ellipsoids [symmetry codes: (i) 1/2-x, -1/2+y, 1/2-z; (ii) x, y, 1+z; (iii) 1-x, 1-y, 1-z; (iv) 1/2-x, -1/2+y, 3/2-z].

**Fig. 2.**
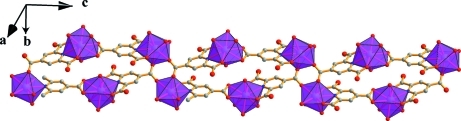
The one-dimensional chain with the rectangular and 8-membered rhombic rings.

**Fig. 3.**
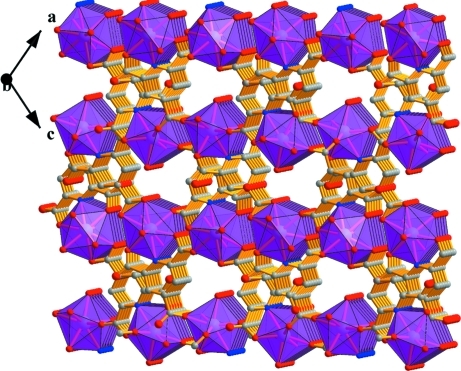
The three-dimensional metal-organic framework in the title compound.

## supplementary crystallographic information

### Comment

In recent years, research of lanthanide pyridine-2,4,6-tricarboxylate coordination polymers has been of great interest in the fields of molecule adsorption, host-guest interaction and luminescence materials (Gao *et al.*, 2006; Li *et al.*, 2008; Wang *et al.*, 2007;) *etc*. The compound {[Pr(H_2_O)_2_(ptc)].2H_2_O} reported by Ghosh *et al.* (2005) represents an example of three-dimensional MOF, which could potentially be utilized as an adsorption material. In this contribution, we report the structure of the title compound.

The asymmetric unit of title compound contains two Sm^III^ ions (Sm1, Sm2), two ptc ligands (denoted as ptc1 and ptc2 ligands, which contain N1 and N2 atoms, respectively) (H_3_ptc = pyridine-2,4,6-tricarboxylate), five aqua ligands and one lattice water molecule as illustrated in Fig. 1. The Sm1 and Sm2 are respectively 9- and 8-coordinated with the ligating atoms occupying the corners of distorted monocapped square anti-prism and 4,4'-bicapped trigonal anti-prism, respectively. The bond distances about the Sm1 and Sm2 atoms fall in the regions 2.406-2.587 Å and 2.359-2.529 Å, the corresponding bond angles are in the regions 50.9-147.8° and 63.3-159.7°, respectively.

Each ptc1 ligand links four Sm^III^ centers with the 4-carboxylate bonded to two metal ions in *syn-syn* bridging fashion and the 2-carboxylate coordinated to two metal ions in *anti-syn* bridging fashion, while each ptc2 ligand connects three Sm^III^ centers with the 4-carboxylate chelating one metal ion and the 2-carboxylate bridging two metal ions in *anti-anti* bridging fashions.

Both ptc1 and ptc2 ligands coordinate Sm1 atoms to form a linear [Sm(ptc)_2_] metallo-ligand, which, in turn, bridges the Sm2 atoms to generate one-dimensional ribbon-like chains with rectangular and 8-membered rhombic rings alternating (Fig. 2). Within the rectangular ring, the two adjacent ptc1 and ptc2 ligands orientate approximately parallelly to each other with a dihedral angle of 7.4° and the mean interplanar distance of 3.33 Å suggests significant intrachain π···π stacking interactions. The resulting one-dimensional chains donate carboxylate O atoms (O1 and O10) to coordinate with Sm atoms from two neighboring chains to construct a three-dimensional framework (Fig. 3).

### Experimental

All chemicals were obtained from commerical sources and were used as obtained. A mixture of SmCl_3_.nH_2_O (0.60 mmol), pyridine-2,4,6-tricarboxylic acid (0.0537 g, 0.25 mmol), Malonic acid (0.0261 g, 0.25 mmol), NaOH (1 ml, 1 *M*) and H_2_O (20 ml) was sealed into a 23 ml Teflon-lined stainless autoclave, which was heated up to 180°C, at which temperature the reactor was held for 3 days, and then cooled to room temperature. A mixture of brownish deposit and a small amount of colourless block-liked crystals were obtained.

### Refinement

H atoms bonded to C atoms were palced in geometrically calculated position, and were refined using a riding model, with *U*_iso_(H) = 1.2 *U*_eq_(C). H atoms attached to O atoms were found in a difference Fourier synthesis and were refined using a riding model, with the O–H distances fixed as initially found and with *U*_iso_(H) values set at 1.2 *U*eq(O).

### Crystal data

**Table:**

[Sm_2_(C_8_H_2_NO_6_)_2_(H_2_O)_5_]·H_2_O	*F*(000) = 1576
*M**_r_* = 825.01	*D*_x_ = 2.498 Mg m^−^^3^
Monoclinic, *P*2_1_/*n*	Mo *K*α radiation, λ = 0.71073 Å
Hall symbol: -P 2yn	Cell parameters from 19369 reflections
*a* = 18.426 (4) Å	θ = 3.2–27.5°
*b* = 6.9082 (14) Å	µ = 5.40 mm^−^^1^
*c* = 18.583 (4) Å	*T* = 293 K
β = 111.98 (3)°	Block, colorless
*V* = 2193.6 (8) Å^3^	0.43 × 0.28 × 0.21 mm
*Z* = 4	

### Data collection

**Table:**

Rigaku R-AXIS RAPID diffractometer	4963 independent reflections
Radiation source: fine-focus sealed tube	4776 reflections with *I* > 2σ(*I*)
Graphite monochromator	*R*_int_ = 0.035
Detector resolution: 0 pixels mm^-1^	θ_max_ = 27.5°, θ_min_ = 3.2°
ω scans	*h* = −23→23
Absorption correction: multi-scan (*ABSCOR*; Higashi, 1995)	*k* = −8→8
*T*_min_ = 0.177, *T*_max_ = 0.321	*l* = −24→22
20300 measured reflections	

### Refinement

**Table:**

Refinement on *F*^2^	Secondary atom site location: difference Fourier map
Least-squares matrix: full	Hydrogen site location: inferred from neighbouring sites
*R*[*F*^2^ > 2σ(*F*^2^)] = 0.018	H-atom parameters constrained
*wR*(*F*^2^) = 0.041	*w* = 1/[σ^2^(*F*_o_^2^) + (0.0066*P*)^2^ + 3.5019*P*] where *P* = (*F*_o_^2^ + 2*F*_c_^2^)/3
*S* = 1.19	(Δ/σ)_max_ = 0.002
4963 reflections	Δρ_max_ = 0.69 e Å^−^^3^
344 parameters	Δρ_min_ = −0.84 e Å^−^^3^
0 restraints	Extinction correction: *SHELXL97* (Sheldrick, 2008), Fc^*^=kFc[1+0.001xFc^2^λ^3^/sin(2θ)]^-1/4^
Primary atom site location: structure-invariant direct methods	Extinction coefficient: 0.00244 (6)

### Special details

**Table:**

Geometry. All e.s.d.'s (except the e.s.d. in the dihedral angle between two l.s. planes) are estimated using the full covariance matrix. The cell e.s.d.'s are taken into account individually in the estimation of e.s.d.'s in distances, angles and torsion angles; correlations between e.s.d.'s in cell parameters are only used when they are defined by crystal symmetry. An approximate (isotropic) treatment of cell e.s.d.'s is used for estimating e.s.d.'s involving l.s. planes.
Refinement. Refinement of *F*^2^ against ALL reflections. The weighted *R*-factor *wR* and goodness of fit *S* are based on *F*^2^, conventional *R*-factors *R* are based on *F*, with *F* set to zero for negative *F*^2^. The threshold expression of *F*^2^ > σ(*F*^2^) is used only for calculating *R*-factors(gt) *etc*. and is not relevant to the choice of reflections for refinement. *R*-factors based on *F*^2^ are statistically about twice as large as those based on *F*, and *R*- factors based on ALL data will be even larger.

### Fractional atomic coordinates and isotropic or equivalent isotropic displacement parameters (Å^2^)

**Table:**

	*x*	*y*	*z*	*U*_iso_*/*U*_eq_	
Sm1	0.373309 (7)	0.222784 (18)	0.378119 (7)	0.01049 (4)	
Sm2	0.374965 (8)	0.250031 (17)	0.896720 (7)	0.01111 (5)	
N1	0.42996 (13)	0.3322 (3)	0.27802 (12)	0.0125 (4)	
C1	0.38355 (15)	0.4105 (3)	0.21147 (15)	0.0127 (5)	
C2	0.40694 (15)	0.4323 (3)	0.14877 (15)	0.0139 (5)	
H2A	0.3735	0.4861	0.1023	0.017*	
C3	0.48176 (15)	0.3710 (3)	0.15774 (15)	0.0125 (5)	
C4	0.53246 (15)	0.3029 (4)	0.22948 (15)	0.0136 (5)	
H4A	0.5840	0.2707	0.2377	0.016*	
C5	0.50357 (15)	0.2846 (3)	0.28859 (15)	0.0119 (5)	
C6	0.30319 (15)	0.4640 (4)	0.20964 (15)	0.0145 (5)	
O1	0.26044 (11)	0.5719 (3)	0.15807 (11)	0.0185 (4)	
O2	0.28618 (11)	0.3912 (3)	0.26427 (11)	0.0206 (4)	
C7	0.50797 (15)	0.3719 (3)	0.08959 (14)	0.0124 (5)	
O3	0.45938 (12)	0.3160 (3)	0.02671 (11)	0.0219 (4)	
O4	0.57737 (11)	0.4236 (3)	0.10278 (11)	0.0172 (4)	
C8	0.55086 (16)	0.2082 (4)	0.36836 (16)	0.0146 (5)	
O5	0.62134 (12)	0.1852 (3)	0.38807 (12)	0.0278 (5)	
O6	0.51110 (11)	0.1716 (3)	0.41069 (11)	0.0183 (4)	
O7	0.36682 (12)	−0.0503 (3)	0.29113 (11)	0.0220 (4)	
H7B	0.3178	−0.0743	0.2654	0.026*	
H7A	0.3869	−0.0502	0.2585	0.026*	
O8	0.40849 (13)	0.5640 (3)	0.39790 (13)	0.0267 (5)	
H8A	0.4563	0.6135	0.4218	0.032*	
H8B	0.3682	0.5986	0.4083	0.032*	
O9	0.37653 (13)	−0.0753 (3)	0.45208 (12)	0.0232 (4)	
H9A	0.4159	−0.0992	0.4946	0.028*	
H9B	0.3363	−0.1166	0.4672	0.028*	
N2	0.38147 (13)	0.2822 (3)	0.76366 (13)	0.0126 (4)	
C9	0.31935 (15)	0.3494 (3)	0.70511 (15)	0.0136 (5)	
C10	0.31717 (16)	0.3588 (4)	0.62931 (15)	0.0164 (5)	
H10A	0.2726	0.4017	0.5888	0.020*	
C11	0.38335 (16)	0.3023 (4)	0.61614 (15)	0.0144 (5)	
C12	0.44856 (17)	0.2360 (3)	0.67751 (16)	0.0142 (5)	
H12A	0.4938	0.2004	0.6699	0.017*	
C13	0.44461 (16)	0.2242 (3)	0.75073 (16)	0.0135 (5)	
C14	0.25231 (16)	0.4146 (4)	0.72805 (15)	0.0148 (5)	
O10	0.19320 (12)	0.4815 (3)	0.67583 (11)	0.0210 (4)	
O11	0.26175 (12)	0.3950 (3)	0.79795 (11)	0.0232 (4)	
C15	0.38312 (17)	0.3038 (4)	0.53467 (15)	0.0157 (5)	
O12	0.44658 (13)	0.2832 (3)	0.52504 (12)	0.0221 (4)	
O13	0.31898 (12)	0.3173 (3)	0.47848 (11)	0.0212 (4)	
C16	0.50960 (15)	0.1461 (3)	0.82233 (15)	0.0134 (5)	
O14	0.57112 (12)	0.0880 (3)	0.81788 (11)	0.0206 (4)	
O15	0.49458 (12)	0.1467 (3)	0.88390 (11)	0.0198 (4)	
O16	0.39568 (13)	−0.0498 (3)	0.97353 (11)	0.0244 (4)	
H16B	0.4251	−0.0676	1.0162	0.029*	
H16A	0.3898	−0.1564	0.9530	0.029*	
O17	0.28096 (12)	0.3100 (3)	0.95460 (13)	0.0283 (5)	
H17B	0.2358	0.3132	0.9261	0.034*	
H17A	0.2806	0.2465	0.9929	0.034*	
O18	0.21204 (15)	0.6072 (3)	0.41898 (14)	0.0374 (6)	
H18B	0.2389	0.5106	0.4367	0.045*	
H18A	0.1688	0.5550	0.3908	0.045*	

### Atomic displacement parameters (Å^2^)

**Table:**

	*U*^11^	*U*^22^	*U*^33^	*U*^12^	*U*^13^	*U*^23^
Sm1	0.00985 (8)	0.01369 (7)	0.00821 (7)	0.00008 (4)	0.00368 (6)	0.00015 (4)
Sm2	0.00839 (8)	0.01720 (7)	0.00731 (7)	−0.00064 (4)	0.00243 (6)	−0.00005 (4)
N1	0.0109 (11)	0.0167 (10)	0.0101 (10)	0.0002 (8)	0.0040 (9)	0.0003 (8)
C1	0.0095 (12)	0.0143 (11)	0.0133 (12)	0.0001 (9)	0.0032 (10)	0.0017 (9)
C2	0.0132 (13)	0.0169 (11)	0.0101 (12)	−0.0002 (9)	0.0026 (10)	0.0028 (9)
C3	0.0128 (13)	0.0136 (11)	0.0118 (12)	−0.0032 (9)	0.0055 (10)	−0.0006 (9)
C4	0.0108 (13)	0.0162 (11)	0.0144 (13)	−0.0015 (9)	0.0055 (11)	−0.0011 (10)
C5	0.0115 (13)	0.0128 (10)	0.0111 (12)	−0.0005 (9)	0.0041 (11)	0.0002 (9)
C6	0.0113 (13)	0.0184 (12)	0.0133 (12)	0.0010 (9)	0.0042 (10)	−0.0015 (10)
O1	0.0137 (10)	0.0243 (9)	0.0164 (10)	0.0060 (7)	0.0045 (8)	0.0061 (8)
O2	0.0128 (10)	0.0339 (10)	0.0171 (10)	0.0053 (8)	0.0079 (8)	0.0105 (8)
C7	0.0137 (13)	0.0127 (10)	0.0105 (12)	0.0009 (9)	0.0044 (10)	0.0024 (9)
O3	0.0208 (11)	0.0313 (10)	0.0112 (10)	−0.0046 (8)	0.0031 (9)	−0.0038 (8)
O4	0.0150 (10)	0.0224 (9)	0.0158 (9)	−0.0015 (7)	0.0077 (8)	0.0010 (7)
C8	0.0127 (13)	0.0158 (11)	0.0140 (13)	0.0001 (9)	0.0036 (11)	−0.0002 (10)
O5	0.0108 (11)	0.0466 (13)	0.0235 (12)	0.0054 (9)	0.0035 (9)	0.0103 (10)
O6	0.0137 (10)	0.0295 (10)	0.0121 (9)	0.0051 (8)	0.0053 (8)	0.0073 (8)
O7	0.0143 (10)	0.0353 (11)	0.0188 (10)	−0.0037 (8)	0.0090 (9)	−0.0115 (9)
O8	0.0268 (12)	0.0190 (9)	0.0329 (12)	−0.0045 (8)	0.0098 (10)	−0.0050 (8)
O9	0.0273 (12)	0.0248 (10)	0.0169 (10)	0.0035 (8)	0.0074 (9)	0.0062 (8)
N2	0.0114 (11)	0.0163 (9)	0.0095 (11)	0.0000 (8)	0.0033 (9)	0.0004 (8)
C9	0.0136 (13)	0.0157 (11)	0.0113 (12)	0.0018 (9)	0.0044 (10)	0.0009 (9)
C10	0.0168 (14)	0.0197 (12)	0.0111 (13)	0.0039 (10)	0.0034 (11)	0.0030 (10)
C11	0.0173 (14)	0.0152 (11)	0.0114 (13)	0.0001 (9)	0.0062 (11)	−0.0017 (10)
C12	0.0137 (14)	0.0169 (12)	0.0128 (13)	0.0007 (9)	0.0058 (12)	−0.0007 (9)
C13	0.0131 (14)	0.0140 (11)	0.0133 (13)	−0.0002 (9)	0.0049 (11)	−0.0013 (9)
C14	0.0139 (13)	0.0171 (11)	0.0126 (12)	0.0028 (9)	0.0039 (10)	0.0006 (10)
O10	0.0170 (11)	0.0284 (10)	0.0149 (10)	0.0110 (8)	0.0029 (8)	0.0043 (8)
O11	0.0167 (11)	0.0417 (12)	0.0124 (10)	0.0096 (9)	0.0067 (9)	0.0040 (8)
C15	0.0216 (14)	0.0164 (11)	0.0105 (13)	0.0019 (10)	0.0076 (11)	−0.0003 (10)
O12	0.0175 (11)	0.0370 (11)	0.0136 (10)	0.0003 (8)	0.0077 (9)	−0.0027 (8)
O13	0.0180 (11)	0.0347 (11)	0.0099 (9)	0.0068 (8)	0.0042 (8)	−0.0007 (8)
C16	0.0129 (13)	0.0135 (11)	0.0132 (12)	−0.0016 (9)	0.0042 (10)	0.0001 (9)
O14	0.0137 (10)	0.0303 (10)	0.0194 (10)	0.0067 (8)	0.0079 (9)	0.0014 (8)
O15	0.0152 (10)	0.0336 (10)	0.0113 (9)	0.0068 (8)	0.0057 (8)	0.0043 (8)
O16	0.0297 (12)	0.0225 (10)	0.0140 (10)	0.0053 (8)	0.0002 (9)	0.0034 (8)
O17	0.0111 (11)	0.0532 (13)	0.0215 (11)	0.0062 (9)	0.0071 (9)	0.0058 (10)
O18	0.0347 (15)	0.0383 (13)	0.0323 (13)	0.0133 (10)	0.0046 (11)	−0.0015 (10)

### Geometric parameters (Å, º)

**Table:**

Sm1—O6	2.406 (2)	O3—Sm2^vi^	2.377 (2)
Sm1—O2	2.422 (2)	O4—Sm2^iv^	2.4185 (18)
Sm1—O8	2.436 (2)	C8—O5	1.221 (3)
Sm1—O7	2.4582 (19)	C8—O6	1.285 (3)
Sm1—O9	2.4642 (19)	O7—H7B	0.8654
Sm1—O13	2.5110 (18)	O7—H7A	0.8193
Sm1—O1^i^	2.5243 (19)	O8—H8A	0.8930
Sm1—N1	2.564 (2)	O8—H8B	0.8667
Sm1—O12	2.587 (2)	O9—H9A	0.8662
Sm1—C15	2.901 (3)	O9—H9B	0.9304
Sm2—O10^ii^	2.3600 (19)	N2—C9	1.333 (3)
Sm2—O3^iii^	2.377 (2)	N2—C13	1.335 (3)
Sm2—O17	2.3925 (19)	C9—C10	1.396 (3)
Sm2—O15	2.4120 (19)	C9—C14	1.518 (3)
Sm2—O4^iv^	2.4185 (19)	C10—C11	1.387 (3)
Sm2—O11	2.420 (2)	C10—H10A	0.9300
Sm2—O16	2.463 (2)	C11—C12	1.389 (4)
Sm2—N2	2.530 (2)	C11—C15	1.512 (3)
N1—C1	1.327 (3)	C12—C13	1.392 (4)
N1—C5	1.337 (3)	C12—H12A	0.9300
C1—C2	1.394 (3)	C13—C16	1.518 (4)
C1—C6	1.514 (3)	C14—O10	1.245 (3)
C2—C3	1.391 (4)	C14—O11	1.252 (3)
C2—H2A	0.9300	O10—Sm2^vii^	2.3600 (19)
C3—C4	1.393 (4)	C15—O13	1.254 (3)
C3—C7	1.515 (3)	C15—O12	1.256 (3)
C4—C5	1.394 (3)	C16—O14	1.234 (3)
C4—H4A	0.9300	C16—O15	1.274 (3)
C5—C8	1.506 (4)	O16—H16B	0.7852
C6—O1	1.237 (3)	O16—H16A	0.8179
C6—O2	1.273 (3)	O17—H17B	0.8023
O1—Sm1^v^	2.5243 (19)	O17—H17A	0.8383
C7—O3	1.238 (3)	O18—H18B	0.8223
C7—O4	1.260 (3)	O18—H18A	0.8524
			
O6—Sm1—O2	125.63 (6)	C2—C1—C6	124.1 (2)
O6—Sm1—O8	84.72 (7)	C3—C2—C1	118.0 (2)
O2—Sm1—O8	73.68 (8)	C3—C2—H2A	121.0
O6—Sm1—O7	80.78 (7)	C1—C2—H2A	121.0
O2—Sm1—O7	86.62 (7)	C2—C3—C4	119.8 (2)
O8—Sm1—O7	141.88 (7)	C2—C3—C7	120.7 (2)
O6—Sm1—O9	86.22 (7)	C4—C3—C7	119.5 (2)
O2—Sm1—O9	139.32 (7)	C3—C4—C5	117.9 (2)
O8—Sm1—O9	140.81 (7)	C3—C4—H4A	121.1
O7—Sm1—O9	73.17 (7)	C5—C4—H4A	121.1
O6—Sm1—O13	121.87 (7)	N1—C5—C4	121.8 (2)
O2—Sm1—O13	101.87 (6)	N1—C5—C8	114.3 (2)
O8—Sm1—O13	78.10 (7)	C4—C5—C8	123.8 (2)
O7—Sm1—O13	138.87 (7)	O1—C6—O2	125.5 (2)
O9—Sm1—O13	74.70 (7)	O1—C6—C1	119.9 (2)
O6—Sm1—O1^i^	147.10 (6)	O2—C6—C1	114.6 (2)
O2—Sm1—O1^i^	72.76 (7)	C6—O1—Sm1^v^	136.93 (16)
O8—Sm1—O1^i^	128.16 (7)	C6—O2—Sm1	127.30 (17)
O7—Sm1—O1^i^	72.87 (6)	O3—C7—O4	126.5 (2)
O9—Sm1—O1^i^	67.72 (7)	O3—C7—C3	116.3 (2)
O13—Sm1—O1^i^	71.60 (7)	O4—C7—C3	117.1 (2)
O6—Sm1—N1	63.12 (7)	C7—O3—Sm2^vi^	170.38 (18)
O2—Sm1—N1	62.70 (7)	C7—O4—Sm2^iv^	127.27 (16)
O8—Sm1—N1	70.53 (7)	O5—C8—O6	125.2 (3)
O7—Sm1—N1	71.46 (6)	O5—C8—C5	120.0 (2)
O9—Sm1—N1	136.02 (6)	O6—C8—C5	114.8 (2)
O13—Sm1—N1	147.78 (7)	C8—O6—Sm1	127.38 (17)
O1^i^—Sm1—N1	123.39 (7)	Sm1—O7—H7B	107.1
O6—Sm1—O12	70.93 (7)	Sm1—O7—H7A	124.1
O2—Sm1—O12	139.30 (7)	H7B—O7—H7A	104.9
O8—Sm1—O12	71.36 (7)	Sm1—O8—H8A	127.1
O7—Sm1—O12	134.07 (7)	Sm1—O8—H8B	95.7
O9—Sm1—O12	69.64 (7)	H8A—O8—H8B	123.4
O13—Sm1—O12	50.94 (7)	Sm1—O9—H9A	120.4
O1^i^—Sm1—O12	114.82 (7)	Sm1—O9—H9B	124.7
N1—Sm1—O12	121.65 (7)	H9A—O9—H9B	99.0
O6—Sm1—C15	96.45 (8)	C9—N2—C13	119.8 (2)
O2—Sm1—C15	123.75 (7)	C9—N2—Sm2	119.21 (16)
O8—Sm1—C15	75.45 (7)	C13—N2—Sm2	120.87 (18)
O7—Sm1—C15	140.98 (7)	N2—C9—C10	122.1 (2)
O9—Sm1—C15	67.82 (7)	N2—C9—C14	114.5 (2)
O13—Sm1—C15	25.51 (7)	C10—C9—C14	123.4 (2)
O1^i^—Sm1—C15	92.01 (7)	C11—C10—C9	118.0 (2)
N1—Sm1—C15	141.37 (7)	C11—C10—H10A	121.0
O12—Sm1—C15	25.64 (7)	C9—C10—H10A	121.0
O10^ii^—Sm2—O3^iii^	137.50 (7)	C10—C11—C12	119.7 (2)
O10^ii^—Sm2—O17	94.18 (8)	C10—C11—C15	120.1 (2)
O3^iii^—Sm2—O17	79.54 (8)	C12—C11—C15	120.1 (2)
O10^ii^—Sm2—O15	91.23 (7)	C11—C12—C13	118.5 (2)
O3^iii^—Sm2—O15	83.25 (7)	C11—C12—H12A	120.8
O17—Sm2—O15	159.69 (7)	C13—C12—H12A	120.8
O10^ii^—Sm2—O4^iv^	148.20 (7)	N2—C13—C12	121.8 (3)
O3^iii^—Sm2—O4^iv^	73.67 (7)	N2—C13—C16	113.8 (2)
O17—Sm2—O4^iv^	99.20 (7)	C12—C13—C16	124.4 (2)
O15—Sm2—O4^iv^	86.13 (7)	O10—C14—O11	126.2 (2)
O10^ii^—Sm2—O11	76.61 (8)	O10—C14—C9	117.2 (2)
O3^iii^—Sm2—O11	137.89 (7)	O11—C14—C9	116.6 (2)
O17—Sm2—O11	72.89 (7)	C14—O10—Sm2^vii^	149.34 (19)
O15—Sm2—O11	127.43 (6)	C14—O11—Sm2	125.26 (16)
O4^iv^—Sm2—O11	79.98 (7)	O13—C15—O12	121.9 (2)
O10^ii^—Sm2—O16	66.67 (7)	O13—C15—C11	118.9 (2)
O3^iii^—Sm2—O16	70.83 (7)	O12—C15—C11	119.2 (3)
O17—Sm2—O16	82.31 (7)	O13—C15—Sm1	59.59 (13)
O15—Sm2—O16	81.90 (7)	O12—C15—Sm1	63.06 (14)
O4^iv^—Sm2—O16	143.57 (7)	C11—C15—Sm1	168.04 (17)
O11—Sm2—O16	133.64 (7)	C15—O12—Sm1	91.30 (17)
O10^ii^—Sm2—N2	73.77 (7)	C15—O13—Sm1	94.91 (15)
O3^iii^—Sm2—N2	136.39 (7)	O14—C16—O15	125.1 (3)
O17—Sm2—N2	137.00 (8)	O14—C16—C13	120.0 (2)
O15—Sm2—N2	63.27 (7)	O15—C16—C13	114.9 (2)
O4^iv^—Sm2—N2	76.81 (6)	C16—O15—Sm2	127.11 (17)
O11—Sm2—N2	64.22 (7)	Sm2—O16—H16B	128.1
O16—Sm2—N2	125.92 (7)	Sm2—O16—H16A	121.5
C1—N1—C5	120.1 (2)	H16B—O16—H16A	104.5
C1—N1—Sm1	120.05 (16)	Sm2—O17—H17B	117.1
C5—N1—Sm1	119.15 (16)	Sm2—O17—H17A	121.9
N1—C1—C2	122.0 (2)	H17B—O17—H17A	103.8
N1—C1—C6	113.8 (2)	H18B—O18—H18A	100.8

Symmetry codes: (i) −*x*+1/2, *y*−1/2, −*z*+1/2; (ii) −*x*+1/2, *y*−1/2, −*z*+3/2; (iii) *x*, *y*, *z*+1; (iv) −*x*+1, −*y*+1, −*z*+1; (v) −*x*+1/2, *y*+1/2, −*z*+1/2; (vi) *x*, *y*, *z*−1; (vii) −*x*+1/2, *y*+1/2, −*z*+3/2.

### Hydrogen-bond geometry (Å, º)

**Table:**

*D*—H···*A*	*D*—H	H···*A*	*D*···*A*	*D*—H···*A*
O7—H7*A*···O14^viii^	0.82	1.87	2.686 (3)	172
O7—H7*B*···O2^i^	0.86	1.80	2.645 (3)	165
O8—H8*A*···O12^iv^	0.89	1.83	2.727 (3)	177
O8—H8*B*···O9^ix^	0.87	2.38	2.831 (3)	113
O9—H9*A*···O6^viii^	0.87	1.84	2.698 (3)	171
O9—H9*B*···O5^viii^	0.93	2.55	3.052 (3)	114
O16—H16*A*···O4^viii^	0.82	2.31	3.075 (3)	157
O16—H16*B*···O15^x^	0.79	1.97	2.747 (3)	171
O17—H17*A*···O18^ii^	0.84	1.86	2.697 (3)	176
O17—H17*B*···O5^xi^	0.80	1.96	2.731 (3)	162
O18—H18*A*···O14^xii^	0.85	2.06	2.905 (3)	174
O18—H18*B*···O13	0.82	1.93	2.736 (3)	168

Symmetry codes: (i) −*x*+1/2, *y*−1/2, −*z*+1/2; (ii) −*x*+1/2, *y*−1/2, −*z*+3/2; (iv) −*x*+1, −*y*+1, −*z*+1; (viii) −*x*+1, −*y*, −*z*+1; (ix) *x*, *y*+1, *z*; (x) −*x*+1, −*y*, −*z*+2; (xi) *x*−1/2, −*y*+1/2, *z*+1/2; (xii) *x*−1/2, −*y*+1/2, *z*−1/2.
